# Endoscopic Retrieval of Ingested Bags Following a Multidisciplinary Approach

**DOI:** 10.7759/cureus.8746

**Published:** 2020-06-21

**Authors:** Harman K Rahal, Rani Berry, James H Tabibian

**Affiliations:** 1 Internal Medicine, University of California Los Angeles Medical Center, Los Angeles, USA; 2 Gastroenterology, Olive View-University of California Los Angeles Medical Center, Sylmar, USA; 3 Gastroenterology, David Geffen School of Medicine at University of California Los Angeles, Los Angeles, USA

**Keywords:** foreign body ingestion, endoscopy, gastric outlet obstruction, abdominal pain, multidisciplinary decision-making

## Abstract

Foreign body ingestion encompasses a broad variety of ingested objects, clinical presentations, and treatment approaches, with a wide spectrum of severity and urgency. Herein, we describe the case of a 29-year-old man presenting with abdominal pain following the ingestion of empty plastic bags. Monitoring with serial imaging demonstrated the bags in the stomach 18 hours post-ingestion. Given this finding and worsening pain, a multidisciplinary decision was made to pursue endoscopic retrieval. This case uniquely demonstrates the benefit of rapid multidisciplinary meetings in an emergency room setting leading to the successful removal of ingested bags from the gastric body. While the phenomenon of “body stuffing,” or hasty ingestion of bagged drugs to evade law enforcement has become common, there are few reports of endoscopic removal for such cases or those involving empty bag ingestion. This case highlights the importance of repeat abdominal imaging and early endoscopic intervention for foreign objects such as bags as they may be difficult to visualize on imaging, making it unreliable to track their progress. Dynamic imaging should be obtained, with computed tomography (CT) being the gold standard. This report represents the first case of empty bag ingestion, highlighting tenets of timely multidisciplinary management and considerations in endoscopic retrieval as a minimally invasive technique when a patient presents in the emergency department following bag ingestion.

## Introduction

In the case of known foreign body ingestion, the treatment algorithm is generally straightforward--sharp, magnetized, toxic, or otherwise deleterious objects should be retrieved urgently if not emergently. However, when facing an unknown foreign body ingestion or one not clearly falling into an established foreign body category, the evaluation must be tailored on an ad hoc basis and may benefit from multidisciplinary coordination. 

In the absence of a universal management consensus, published clinical practice guidelines typically favor a conservative approach when the benefits of expeditious retrieval do not clearly outweigh the risks, e.g. monitoring a patient while waiting for spontaneous evacuation, with or without non-stimulant laxative administration. A major challenge, though, is that the risk:benefit profile may be difficult to assess. For example, foreign bodies may unexpectedly become retained in the stomach causing gastric outlet obstruction and/or degrade (potentially releasing hazardous substances), while on the other hand, endoscopic and even more so surgical intervention carries its own risks, including perforation, infection, toxidromes, and need for bowel resection [1,2].

In the present case, we describe the unique scenario of intentional ingestion of reportedly empty plastic bags by a young man brought in from prison. This case is novel in that: i) clinical management of empty bag ingestion has not been described in the literature, thus representing a gap in practice knowledge; 2) it highlights the importance of multidisciplinary care and dynamic assessment in atypical scenarios; and 3) it demonstrates the feasibility of early endoscopic retrieval of foreign bodies that would, under existing guidelines, be considered safe, thus not requiring urgent endoscopy. As such, we believe this report will be a useful guide for clinicians managing foreign body ingestion, and in particular, cases of bag and other ingestions not fitting into established management algorithms. 

## Case presentation

**Figure 1:**
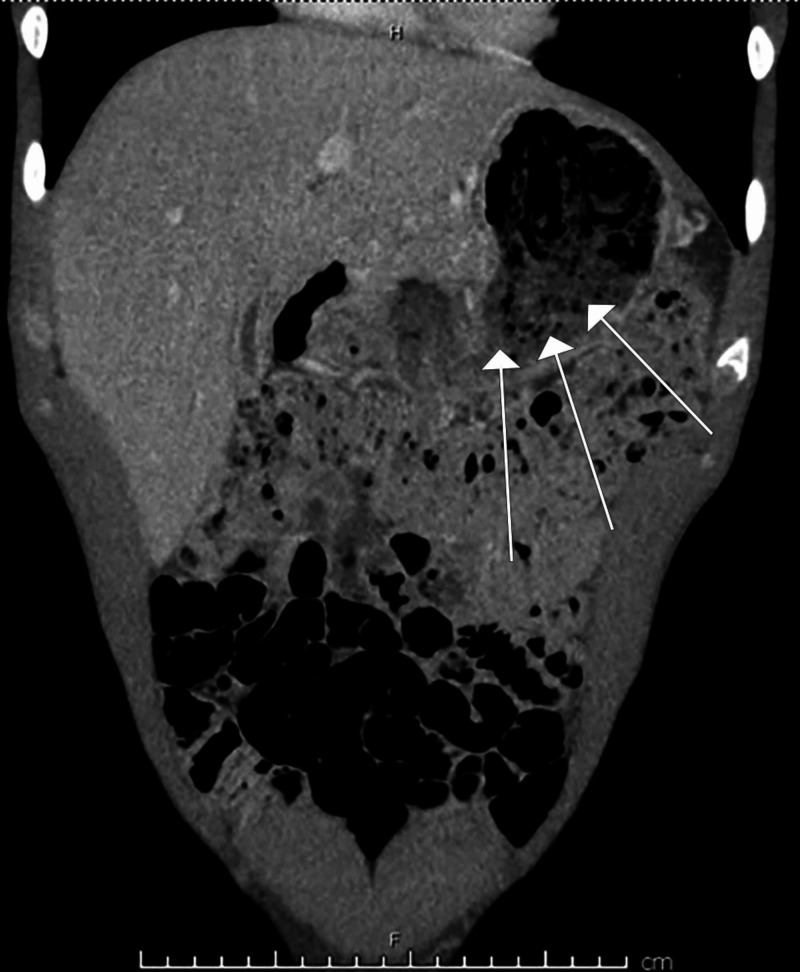
Computed tomography (CT) coronal tomogram demonstrating amorphous material in the stomach compatible with ingested plastic bags (marked with white arrows).

**Figure 2:**
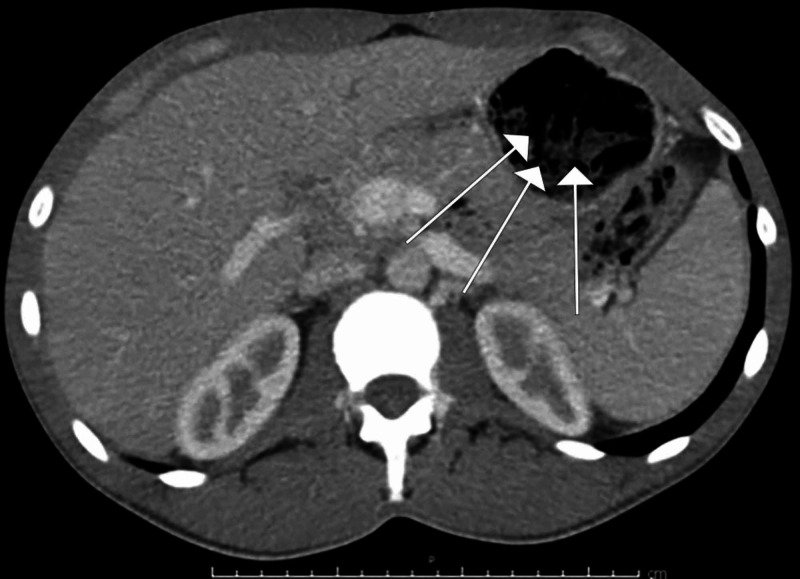
Computed tomography (CT) axial tomogram demonstrating amorphous material in the stomach compatible with ingested plastic bags (marked with white arrows).

**Figure 3:**
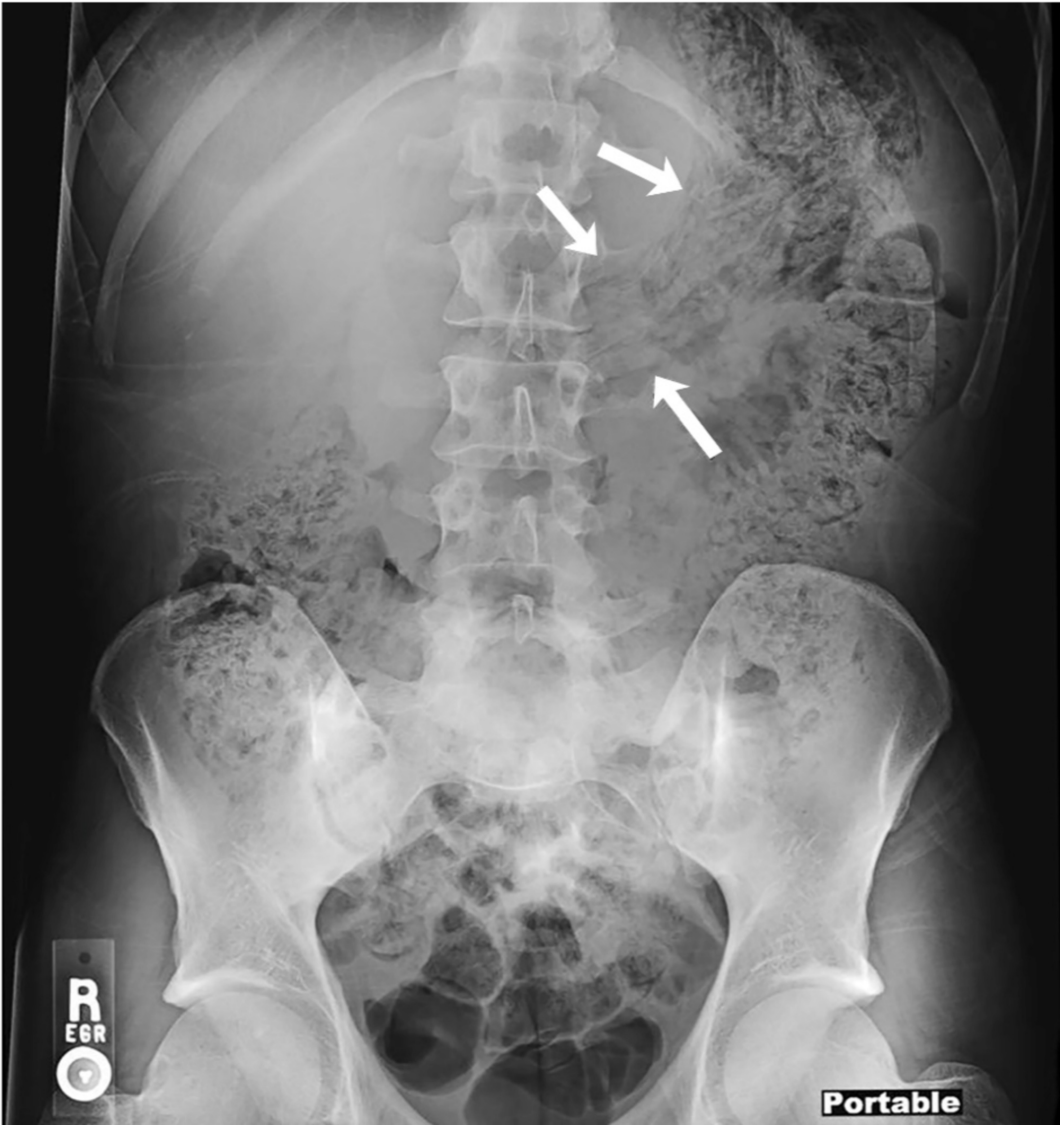
Abdominal X-ray findings demonstrating amorphous material with a linear appearance in the stomach (marked with white arrows).

A 29-year-old male inmate with no significant medical history was brought into the emergency department after ingestion of three large plastic bags following news of life imprisonment. On initial evaluation, vital signs and laboratory tests were normal. Computed tomography (CT) with intravenous contrast was notable for a large amount of amorphous debris in the stomach (Figures 1-2). Given the possibility that the bags would spontaneously pass through the digestive tract, serial abdominal X-rays were ordered to track their transit. An abdominal X-ray one hour later reported no well-defined foreign body. Two hours after initial CT imaging, another abdominal X-ray demonstrated amorphous material with a linear appearance in the stomach, thought to possibly represent ingested foreign bodies (Figure 3). Subsequent imaging ten hours later demonstrated similar findings without passage into the distal small intestine. Imaging was unable to provide definitive evidence that the ingested bags were empty. 

**Figure 4:**
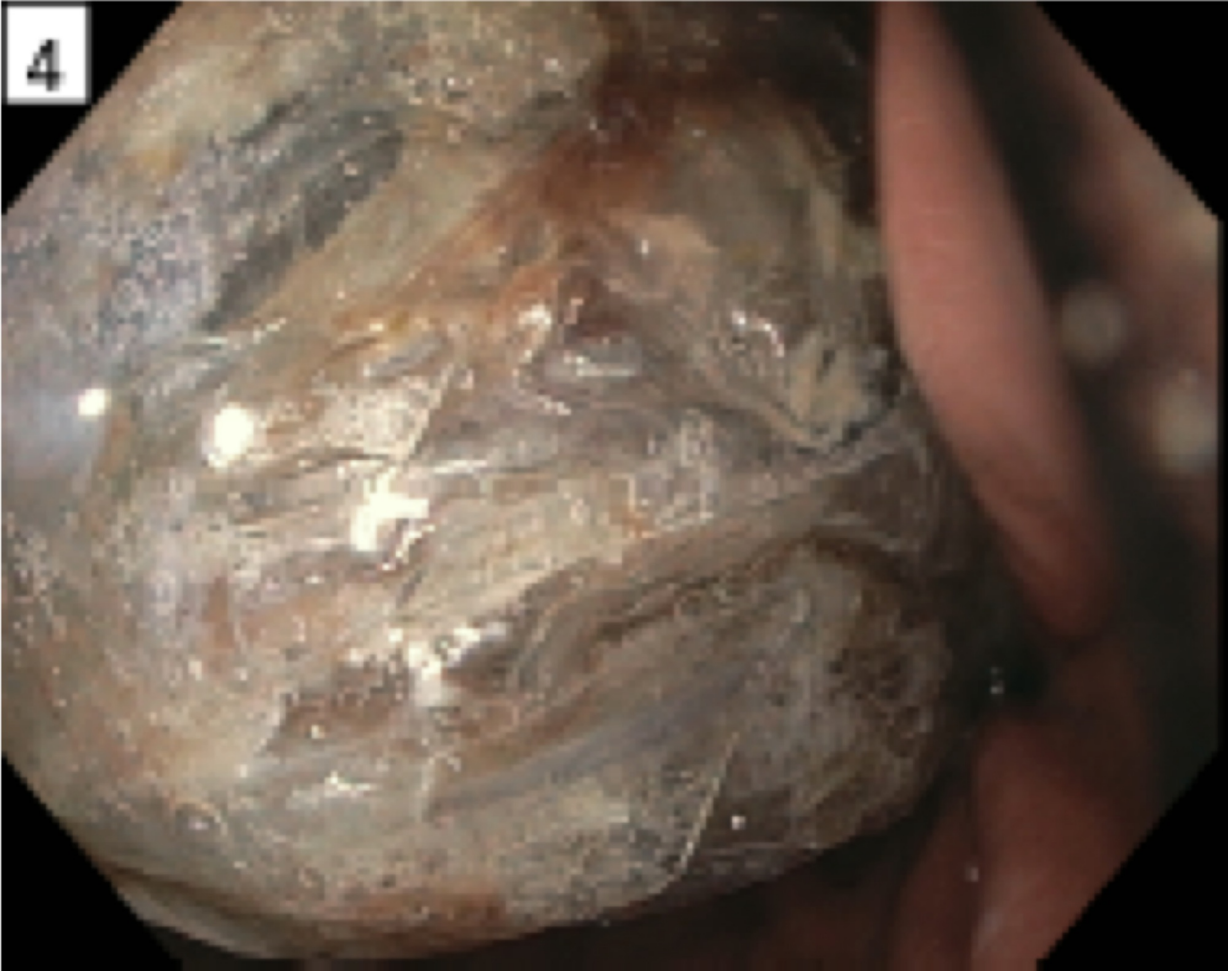
Plastic bags contained in the gastric lumen as visualized upon esophagogastroduodenoscopy.

**Figure 5:**
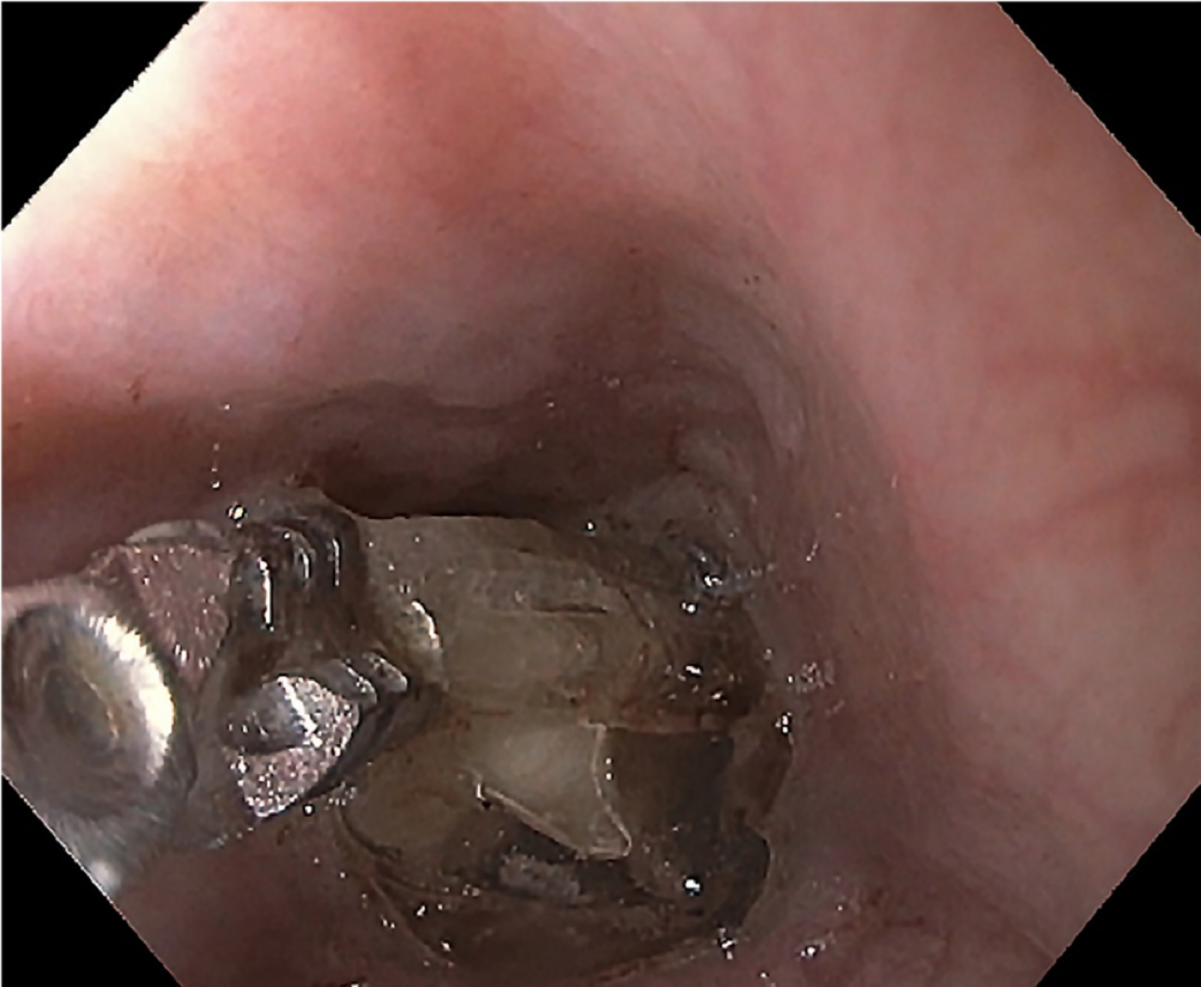
Magnified view of ingested bags being removed endoscopically with rat-tooth forceps.

**Figure 6:**
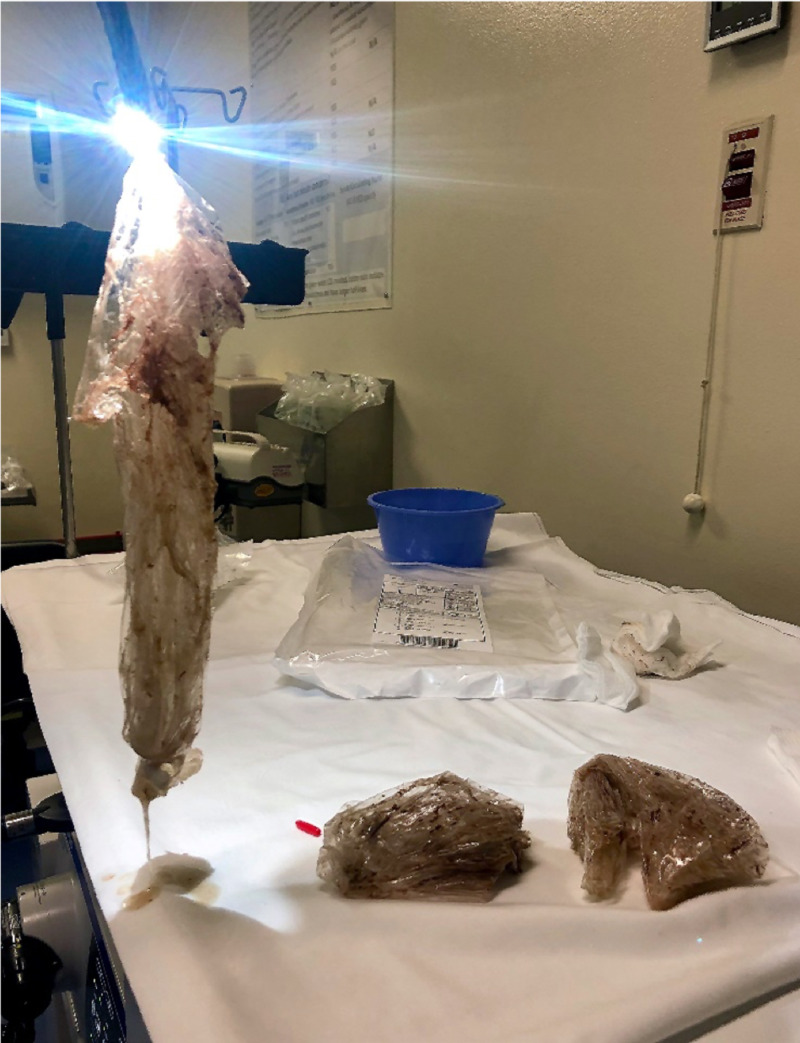
Three large, empty plastic bags sequentially retrieved from the stomach via esophagogastroduodenoscopy.

Given the concern that the bags may contain illicit substances, traditionally regarded as a contraindication to endoscopy, surgery was consulted; however, a multi-specialty (emergency medicine, gastroenterology, surgery, and radiology) decision was made to trial endoscopic retrieval prior to the bags reaching the level of the midgut given the high risk of surgical complications. Esophagogastroduodenoscopy (EGD) showed plastic bags occupying nearly the entire gastric lumen with adherent debris and scattered erosions in the distal gastric body (Figure 4). The gastric mucosa was otherwise normal. Three large plastic bags were retrieved sequentially with rat-tooth forceps (Figures 5-6). A post-procedural abdominal X-ray confirmed complete retrieval.

## Discussion

This case uniquely demonstrates the successful retrieval of ingested bags from the gastric body 18 hours after ingestion. Bag ingestion is a case that is often first encountered in the emergency department. Algorithms exist for ingestions of most known objects, which make triaging in the emergency department much simpler. While the phenomenon of “body stuffing,” or hasty ingestion of bagged drugs to evade law enforcement has become common, there are few published reports of endoscopic retrieval for such cases or those involving empty bag ingestion. This case highlights the importance of repeat abdominal imaging to track progress of foreign objects such as empty bags, with CT being the gold standard given challenges in reliable visualization on X-ray, and consideration of early endoscopic retrieval to mitigate the need for more invasive procedures and potential complications of delayed intervention. 

Endoscopic retrieval of ingested bags has historically not been favored due to the risk of bag rupture, but this has been limited to the context of body packing (i.e. with small sachets of illicit drugs) [3]. Thus, empty bag ingestion represents a different clinical entity not clearly addressed by current guidelines. As illustrated in this case, when the bags have failed to pass to the level of the small intestine, and especially with persistent or increasing symptoms (e.g. abdominal pain, nausea), the risks of bag retention in the gastric antrum and subsequent gastric outlet obstruction should be weighed against the benefits of endoscopic retrieval; in many patients, we believe the latter should be considered and expeditiously pursued in the absence of absolute contraindications. Moreover, even if the bags pass through the pylorus, there is a chance of more distal obstruction at the level of the terminal ileum, at which point endoscopic retrieval may become even more challenging, necessitating surgical intervention. Therefore, we propose that there is a pivotal early window, whilst vital signs are stable and prior to evidence of complications (e.g. perforation, aspiration), during which endoscopic intervention has the highest likelihood of clinical success with an acceptably low risk of adverse events, and that would apply to ingested objects (including but not limited to empty bags) that are not traditionally regarded as urgent scenarios [4,5]. If there is potential that the bags have toxic contents, further risk:benefit assessment and interventional planning may be needed given the possibility of a toxidrome [6].

## Conclusions

Herein, we describe a case of empty bag ingestion, with no previously known algorithms for treatment. Effective diagnosis and consultation for empty bag ingestion can lead to same-day discharges, and rapid treatment for these patients. This case can also support physicians in their discussions with either surgical or gastroenterology services to support their decision making and request for consultation and/or rapid intervention. As evidenced, in patients with foreign body ingestion, the combination of a stable presentation, failure of the ingested material to pass beyond the upper gastrointestinal tract, and/or risk of distal retention, and lack of symptomatic improvement would constitute an indication for timely gastroenterology consultation for endoscopic retrieval. This approach is effective and may prevent complications as well as the need for higher-risk surgical intervention; however, if the bags are known to contain drugs, endoscopic retrieval should be deferred given the risk of bag rupture and systemic toxicity. Though available practice guidelines may recommend elective retrieval for foreign bodies (such as empty bags) which are not sharp, toxic, long, or wide, a coordinated case for urgent endoscopic retrieval can be made and should be considered.
